# ^1^H-NMR metabolomics investigation of CSF from children with HIV reveals altered neuroenergetics due to persistent immune activation

**DOI:** 10.3389/fnins.2024.1270041

**Published:** 2024-04-30

**Authors:** Anicia Thirion, Du Toit Loots, Monray E. Williams, Regan Solomons, Shayne Mason

**Affiliations:** ^1^Department of Biochemistry, Human Metabolomics, Faculty of Natural and Agricultural Sciences, North-West University, Potchefstroom, South Africa; ^2^Department of Pediatrics and Child Health, Faculty of Medicine and Health Sciences, Stellenbosch University, Tygerberg, South Africa

**Keywords:** pediatric, human immunodeficiency virus (HIV), cerebrospinal fluid (CSF), HIV-associated neurocognitive disorder (HAND), proton nuclear magnetic resonance (^1^H-NMR), metabolomics, neuroenergetics

## Abstract

**Background:**

HIV can invade the central nervous system (CNS) early during infection, invading perivascular macrophages and microglia, which, in turn, release viral particles and immune mediators that dysregulate all brain cell types. Consequently, children living with HIV often present with neurodevelopmental delays.

**Methods:**

In this study, we used proton nuclear magnetic resonance (^1^H-NMR) spectroscopy to analyze the neurometabolic profile of HIV infection using cerebrospinal fluid samples obtained from 17 HIV+ and 50 HIV− South African children.

**Results:**

Nine metabolites, including glucose, lactate, glutamine, 1,2-propanediol, acetone, 3-hydroxybutyrate, acetoacetate, 2-hydroxybutyrate, and myo-inositol, showed significant differences when comparing children infected with HIV and those uninfected. These metabolites may be associated with activation of the innate immune response and disruption of neuroenergetics pathways.

**Conclusion:**

These results elucidate the neurometabolic state of children infected with HIV, including upregulation of glycolysis, dysregulation of ketone body metabolism, and elevated reactive oxygen species production. Furthermore, we hypothesize that neuroinflammation alters astrocyte–neuron communication, lowering neuronal activity in children infected with HIV, which may contribute to the neurodevelopmental delay often observed in this population.

## 1 Introduction

Human immunodeficiency virus (HIV)-1 is responsible for the dysregulation of the host immune system and the eventual development of acquired immunodeficiency syndrome (AIDS). Moreover, there are implications of HIV-induced chronic inflammation in the development of several comorbidities that have often been observed in HIV-infected populations, including cardiovascular atherosclerosis, kidney failure, diabetes, cancer, and HIV-associated neurocognitive disorders (HAND) (Cassol et al., 2014; Sailasuta et al., 2016; Kaur et al., 2020). The neuropathogenesis of HIV infection begins with the virus particles entering the central nervous system (CNS) during early infection using infected monocytes and CD4+ T lymphocytes that cross the blood–brain barrier (BBB) and subsequently have detrimental effects both directly due to viral replication and indirectly by dysregulating cellular metabolic processes (González-Scarano and Martín-García, 2005; Smith and Wilkins, 2015; Pajkrt et al., 2016). In the CNS, these infected monocytes differentiate into macrophages, which, in turn, release viral particles that dysregulate all cell types within the CNS compartment as well as virions that infect perivascular macrophages and microglia (González-Scarano and Martín-García, 2005; Smith and Wilkins, 2015). In response, the HIV-infected brain macrophages, microglia, and multinucleated giant cells initiate astrocytosis (Sailasuta et al., 2016). Moreover, HIV induces the activation of host cell metabolism to regulate the adaptive and innate immune responses of the host (Deme et al., 2022). Collectively, these processes contribute to the development and clinical presentation of HAND. Symptoms of HAND may range in severity from asymptomatic neurocognitive impairment (ANI) to minor neurocognitive disorder (MND) and HIV-associated dementia (HAD) (Antinori et al., 2007). HAND is associated with an increased risk for early mortality, independent of clinical predictors (Vazquez-Santiago et al., 2014). While antiretroviral therapy (ART) has reduced the incidence of HIV-induced encephalopathy, 20–50% of children and adults infected with HIV still develop HAND (Cassol et al., 2014; Pajkrt et al., 2016).

In the modern era of ART, there is a link between the aberrations in neuroimmunity and neurometabolic responses in people living with HIV and the development of HAND. In particular, positive feedback loops of immune-mediated tissue damage and repair mechanisms result in chronic inflammation, not entirely corrected with ART (McArthur and Johnson, 2020). The excessive activation of various neuroimmune processes dysregulates normal neurometabolism in the host and results in cellular dysfunction and neurological damage (González-Scarano and Martín-García, 2005; Pajkrt et al., 2016). Interestingly, inflammation can be considered a better predictor for neurocognitive impairment than viral load or CD4+ T-cell counts (Pajkrt et al., 2016). Perinatally infected children are more susceptible to the effects of HIV in the brain, most likely since the time of infection coincides with this critical period of brain development (Schwartz and Major, 2006; Smith and Wilkins, 2015; Pajkrt et al., 2016; Williams et al., 2021b). According to the 2022 UNAIDS report (UNAIDS, 2022), there were approximately 1.7 million children under the age of 15 years living with HIV. Perinatally infected children show neurodevelopmental delay and cognitive impairment, including visual, language, attention, memory, learning, and hearing disabilities (Smith and Wilkins, 2015; Pajkrt et al., 2016; Yadav et al., 2017). Imaging studies indicate that the most common abnormalities in children infected with HIV are calcification of the basal ganglia and frontal periventricular white matter as well as altered cortical thickness and subcortical volumes that are likely due to neuronal and glial cell injury (Smith and Wilkins, 2015; Yadav et al., 2017; McArthur and Johnson, 2020). This injury is likely due to the toxicity of viral proteins, dysregulation of cellular gene expression, and a pro-inflammatory skew, leading to high cerebrospinal fluid (CSF) levels of inflammatory mediators (Smith and Wilkins, 2015; Pajkrt et al., 2016; Yadav et al., 2017; McArthur and Johnson, 2020). There is a potential link between the sustained period of immune activation and neurocognitive damage in both adults and children (Smith and Wilkins, 2015; Williams et al., 2021a).

Considering all of the above, there is limited information regarding the mechanisms of pathogenesis of HIV in the CNS of perinatally infected children. Furthermore, most of the knowledge about potential neurometabolic abnormalities in children has been extrapolated from data collected from adults. The CSF directly interacts with the brain and provides unique insights into the CNS and its conditions. Williams et al. (2021b) conducted a review that examined the association of inflammatory mediators with neurocognitive impairment in children with perinatal HIV infection and found only one study that used CSF samples. Metabolomics is a research approach that generates a more comprehensive characterization of the metabolites within biological samples. This method aims to identify and measure variations in metabolism, providing valuable insights into the changes to biological and cellular processes in response to a perturbation. Untargeted metabolomics is an impartial approach that involves minimal sample preparation, allowing for a comprehensive snapshot of a sample’s metabolome, subsequently identifying disease markers, and providing a better understanding of underlying pathogenic mechanisms. This study aimed to employ proton nuclear magnetic resonance (^1^H-NMR) to generate an untargeted metabolomics dataset to investigate the metabolic underpinnings of HIV-induced neuropathogenesis in the CSF collected from a South African pediatric cohort. Furthermore, we offer new understanding and hypotheses for future investigations in this population.

## 2 Methods and materials

### 2.1 Sample collection

CSF samples were collected by lumbar puncture from 17 HIV+ and 50 HIV- pediatric patients <12 years old (average age [in months] ± standard deviation: HIV+: 29.5 ± 31.2, HIV− 40 ± 32.8) at Tygerberg Hospital in Cape Town, South Africa, in compliance with ethical practices and stored at −80°C. All participants provided written informed consent and/or assent to use their CSF samples for research purposes. The study was approved by the Health Research Ethics Committee (HREC) of Stellenbosch University, Tygerberg Hospital (ethics approval no. N16/11/142), the Western Cape Provincial Government, and the HREC of the North-West University (NWU), Potchefstroom campus (ethics approval no. NWU-00063-18-A1). The frozen CSF samples were transported overnight to the Centre for Human Metabolomics at the Potchefstroom campus of the North-West University, where they were stored in a dedicated −80°C freezer in a Biosafety Level 3 (BSL3) laboratory until analysis. All CSF samples were filtered by centrifugation through Amicon Ultra-2 mL 10,000 MWCO centrifugal filters at 4,500 × *g* for 20 min to remove macromolecular components, such as proteins, and produce a sterile, non-infectious sample for further analysis. Hereafter, 100 μL of the sample was aliquoted for ^1^H-NMR analysis and an additional 20 μL was aliquoted for use in a pooled quality control (QC) sample. ^1^H-NMR was selected as the method of choice because it has a high throughput, utilizes a robust method that provides both quantitative and qualitative data on the metabolic composition of a sample, requires minimal sample preparation (does not chemically alter nor destroy the samples) that contributes toward high repeatability, and allows for the identification of metabolites with a high degree of confidence, with the aid of pure compound spectral libraries and two-dimensional ^1^H-^1^H-NMR data.

### 2.2 Chemicals

Potassium phosphate monobasic (KH_2_PO_4_), sodium azide (NaN_3_), and potassium hydroxide (KOH), were purchased from Sigma-Aldrich (St. Louis, Missouri, United States). Deuterium oxide and trimethylsilyl-2,2,3,3-tetradeuteropropionic acid (TSP) were acquired from Merck (Darmstadt, Germany).

### 2.3 Sample preparation and ^1^H-NMR analysis

An NMR buffer solution was prepared beforehand by dissolving potassium phosphate monobasic (KH_2_PO_4_) in deuterium oxide to a concentration of 1.5 mM. Trimethylsilyl-2,2,3,3-tetradeuteropropionic acid (TSP) was added as a quantitative internal standard. Sodium azide (NaN_3_) dissolved in deuterium oxide was added to prevent bacterial growth. The pH of the solution was adjusted to 7.40 with potassium hydroxide. The standard operating procedure (SOP) used to prepare and analyze the CSF samples on the NMR is outlined in van Zyl et al. (2020), which briefly entails using 54 μL of CSF and 6 μL of NMR buffer in a 2-mm glass NMR tube. Samples were randomized and loaded onto a SampleXpress autosampler with the pooled QC samples placed intermittently, and the analysis was performed at 500 MHz on a Bruker Avance III HD NMR Spectrometer.

Acquisition of ^1^H spectra involved the collection of 128 transients, yielding 32 K data points, utilizing a spectral width of 6,000 Hz, and acquisition time of 2.72 s. The receiver gain was adjusted to 64. The sample temperature was maintained at 300 K, and the H_2_O resonance was suppressed through single-frequency irradiation (NOESY) during a relaxation delay of 4 s, initiated by a 90° excitation pulse lasting 8 μs. Automatic shimming was performed on the deuterium signal. Subsequently, Fourier transformation, phase correction, and baseline correction were carried out automatically. The quality of the spectra was verified by ensuring that the resonance line width for TSP was less than 1 Hz. Bruker Topspin (v3.5) and Bruker AMIX (v3.4.19) were used for data processing, and binning and metabolite identification and quantification, respectively. Selective binning from 0.88 ppm to 5.86 ppm was done by selecting regions with discernible peaks and excluding regions of noise and exogenous compounds such as ethanol (used as a disinfectant during lumbar puncture) and glycerol (a component of the filter membranes) to create a data matrix of 117 bins, excluding the region of the water peak at ~4.7 ppm.

### 2.4 Statistical analysis

Group differences were assessed using the chi-square test. MetaboAnalyst (v5.0) (RRID:SCR_015539) was used for statistical data processing. Bins with QC relative standard deviation values higher than 25%, based on the median absolute deviation, were considered analytically unreliable, and removed. Hereafter, the data were normalized by applying log transformation and auto-scaling. Fold change (FC) analysis with a threshold of > |1.5| was used to compare absolute value changes between the HIV+ and control groups. Principal component analysis (PCA) was performed to visualize the variance between the groups without taking the group classification into account. Partial least squares–discriminant analysis (PLS-DA) was performed to identify the variables that best characterize the differentiated groups when taking group classification into account. PLS-DA variables of importance in projection (VIP) values greater than 1.0 were considered significant. The PLS-DA model was validated by performing a permutation test based on prediction accuracy. The significant bins selected by univariate and multivariate methods were used for metabolite identification by comparing their spectra with a purchased Bruker BioReference spectral library at pH 7.0 and in-house pure compound spectral libraries, after which these metabolites were quantified (μmol/L). IBM SPSS Statistics (v. 28.0.1.1) (RRID:SCR_019096) was used to perform the Mann–Whitney *U*-test (a non-parametric *t*-test, selected due to the relatively small sample size and right-skewed distribution of the data) and determine Cohen’s *d*-value for all these metabolites, with *p* < 0.05 and *d* > |0.5| considered to be significant, respectively. Finally, Spearman’s rho correlations were performed for the HIV+ group using IBM SPSS Statistics to identify significant correlations among the metabolites as well as significant correlations between metabolites and continuous clinical variables, while point-biserial correlation was performed for metabolites and dichotomous clinical variables. Correlations were considered significant at the 0.05 level (two-tailed), corrected for multiple comparisons using FDR.

## 3 Results

### 3.1 Participants

CSF samples were obtained via lumbar puncture for routine diagnostic purposes between 2010 and 2017 from 67 pediatric patients under 12 years of age who were suspected of having meningitis. Of these patients, 50 were HIV negative (controls), while 17 were HIV positive (HIV+). All patients underwent a comprehensive clinical evaluation, and their CSF was examined using various diagnostic techniques, including macroscopic appearance, total and differential cell count, protein, glucose, chloride, Gram stain, India ink examination, auramine “O” fluorescence microscopy, culture for pyogenic bacteria, culture for *Mycobacterium tuberculosis* (M.tb), GenoType MTBDRplus assay, and GeneXpert MTB/RIF^®^ assay. HIV status was determined either by enzyme-linked immunosorbent assay (ELISA) or by HIV DNA polymerase chain reaction (PCR) tests. Of the 17 HIV+ children, 4 were on ART. Moreover, the HIV+ group presented with multiple co-infections, and six had concurrent extra-neural M.tb infection (non-CNS TB). M.tb-positive cases were excluded from the controls to obtain the healthiest possible control group to best characterize the metabolic profile of HIV. With regard to neurological symptoms, there were no significant differences between the HIV+ and control groups (Table 1). For obvious ethical reasons, collecting CSF from healthy children is not a viable option, and this control group, although HIV and TB negative, did present to the clinic/hospital with other neurological complications. Consequently, the metabolites that were found to distinguish between the HIV+ and control groups could not have arisen due to a generic response to neurological injury or neuroinflammation. Thus, the features selected were specific to HIV infection. A summary of the pertinent clinical demographics of the cases used for metabolic profiling in this study is given in Table 1.

**Table 1:** Clinical metadata.

	HIV+ (*N* = 17)	Controls (*N* = 50)	HIV vs. control
Male/female, *n* (%)	12/5 (70.6/29.4)	34/16 (68/32)	*p* = 1.000
**Age in months**			
Average (SD)	29.5 (31.2)	40 (32.8)	*p* = 0.240
**Clinical symptoms, *n* (%)**			
Fever	13 (76.5)	31 (62)	*p* = 0.408
Cough	11 (64.7)	14 (28)	*p* = 0.005*
Vomiting	6 (35.3)	26 (52)	*p* = 0.333
Diarrhea	5 (29.4)	9 (18)	*p* = 0.549
Weight loss	2 (11.8)	5 (10)	*p* = 1.000
Headache	5 (29.4)	12 (24)	*p* = 0.696
Decreased consciousness	2 (11.8)	10 (20)	*p* = 0.235
**Concurrent infections, *n* (%)**			
Pneumonia	5 (29.4)	9 (18)	*p* = 0.242
Urinary tract infection	1 (5.8)	0	*p* = 0.418
Gastroenteritis	2 (11.8)	2 (4)	*p* = 0.418
Varicella zoster infection	0	1 (2)	*p* = 1.000
Flue	0	3 (6)	*p* = 0.621
Tonsillitis	0	3 (6)	*p* = 0.621
Abscesses	0	1 (2)	*p* = 1.000
*M.tb* (non-CNS) infection	7 (41.2)	0	*p* ≤ 0.005*
**Neurological symptoms**			
Seizures	5 (29.4)	18 (36)	*p* = 0.943
Raised intracranial pressure	1 (5.8)	0	*p* = 0.622
Meningeal irritation	3 (17.5)	5 (10)	*p* = 0.311
Brainstem dysfunction	0	1 (2)	*p* = 1.000
Infarction	2 (11.8)	1 (2)	*p* = 0.351
Basal enhancement	0	3 (7)	*p* = 0.684
Hydrocephalus	1 (5.8)	1 (2)	*p* = 1.000
Hemiplegia	2 (11.8)	1 (2)	*p* = 0.071
Abnormal MRI	0	0	
**CSF cell count (cells per μL CSF)**			
Erythrocytes [median (IQR)]	0 (0–115.5)	0 (0–1)	*p* = 0.394
Leukocytes [median (IQR)]	1 (0–2)	0 (0–1)	*p* = 0.947
PMNs [median (IQR)]	0	0	*p* = 0.890
Lymphocytes [median (IQR)]	1 (0–1.5)	0 (0–1)	*p* = 0.975
CSF protein (g/L) [median (IQR)]	0.2 (0.2–0.3)	0.17 (0.14–0.27)	*p* = 0.430
CSF glucose (mmol/L) [median (IQR)]	3.3 (3.2–4)	3.8 (3.4–4.1)	*p* = 0.352
CD4 count (cells/μL) [median (IQR)]	142.5 (10.7–383.8)	N/A	
CD4 count (%) [median (IQR)]	22 (19.5–27)	N/A	
Viral load (copies/mL) [median (IQR)]	250,000 (199,750–250,000)	N/A	
On AART, *n* (%)	4 (23.5)	0	
Details of AART	Kaletra, stavudine, 3TC—duration 4 months		
	Kaletra, abacavir, ritonavir, 3TC—duration 25 days		
	Abacavir, kaletra, 3TC—duration 10 months		
	Lamivudine, abacavir, lopinavir/ritonavir—duration 21 days		

Group differences were assessed using the chi-squared test with *p* < 0.05 considered significant.N, number; SD, standard deviation; IQR, interquartile range; M.tb, *Mycobacterium tuberculosis*; CNS, central nervous system; CSF, cerebrospinal fluid.

### 3.2 Significant bins identified by multivariate and univariate methods

#### 3.2.1 Principal component analysis

**Figure 1:**
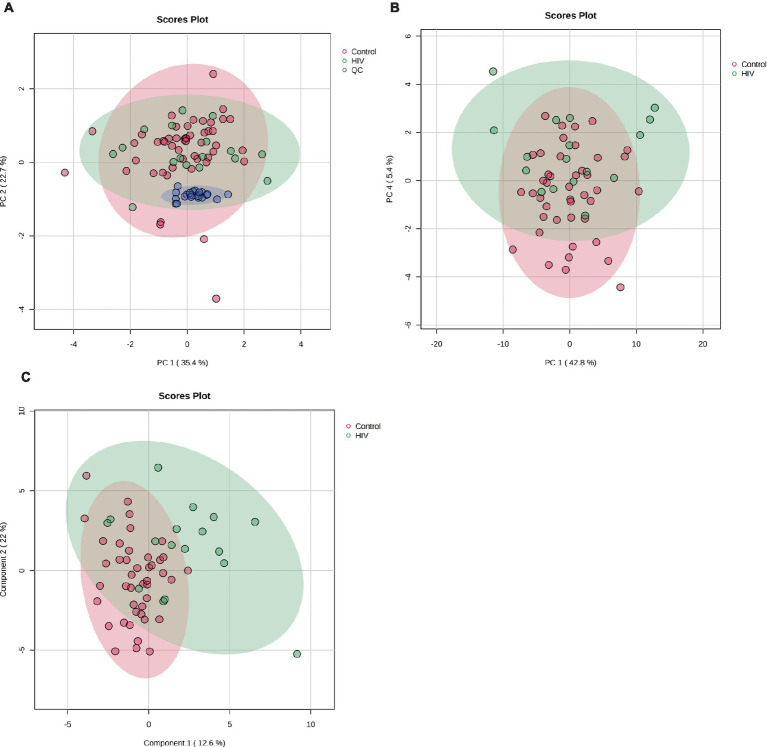
**(A)** PCA scores plot of PC 1 and 2 indicating grouping of QC samples (blue) vs. the 17 HIV+ (green) and 43 control (red) samples. The tighter clustering of the QC samples indicates the repeatability of the analysis. **(B)** PCA scores plot of PC 1 and 4 indicating a partial separation between the 17 HIV+ (green) and 43 control (red) samples. **(C)** PLS-DA scores plot indicating grouping between the 17 HIV+ (green) and 43 control (red) samples.

Figure 1A shows the clustering of the QC and the experimental samples. The scores plot shows that the QC samples cluster closely together, showing far less variation than the patient samples, indicating the precision of the analysis and data collection. Seven control samples appeared outside the PCA 95% confidence interval and were removed as outliers (Puchades-Carrasco et al., 2016). Thus, 43 control samples were compared to 17 HIV+ samples for this analysis. A visual inspection of the PCA scores plot shows a partial separation between PC1 vs. PC4 (Figure 1B) between the HIV+ and control groups, comparatively.

#### 3.2.2 Partial least squares–discriminant analysis

A PLS-DA modeling (Figure 1C) was performed to identify spectral bins that differentiated between the HIV+ and control groups. The PLS-DA model was validated (*p* = 0.0005) by performing a permutation test (*n* = 2,000) based on prediction accuracy, and provided a reasonable measure for the Q2 value, indicating that the PLS-DA model does not suffer from overfitting and the class separation results are based upon predictions, instead of fitted results (Westerhuis et al., 2008). The bins (VIP > 1.0) identified by PLS-DA that are significantly increased in the HIV+ group comparatively include 4.09, 3.61, 4.07, 1.13, 1.35, 3.31, 4.11, 2.07, 1.33, 3.53, 3.29, 2.49, and 4.13. Bins that are significantly decreased in the HIV+ group comparatively are 3.35, 3.25, 3.49, 3.41, 2.39, 3.51, 5.25, 3.39, 3.23, 2.29, 3.43, 3.83, and 0.91.

#### 3.2.3 Univariate analysis

The significant bins (summarized in Table 2), selected by absolute fold change (FC) > |1.5|, which are increased in the HIV+ group compared to the control group, are: 4.09, 3.61, 4.07, 1.13, 3.31, and 3.29, while 2.29 was decreased comparatively. There are, however, no statistically significant bins (*p* < 0.1) based on the Wilcoxon test.

**Table 2:** Summary of statistical findings for binned dataset.

Bins	VIP	FC	Log2(FC)
4.09	2.12	1.72	0.78
3.61	1.83	1.85	0.88
4.07	1.78	1.97	0.98
1.13	1.43	3.03	1.60
1.35	1.54		
3.35	1.31		
3.25	1.36		
3.49	1.32		
3.31	1.27	1.84	0.88
3.41	1.28		
4.11	1.34		
2.07	1.14		
2.39	1.21		
1.33	1.35		
3.51	1.19		
5.25	1.19		
3.39	1.16		
3.53	1.15		
3.23	1.15		
3.29	1.14	1.77	0.82
2.29	1.08	0.36	−1.47
2.49	1.02		
3.43	1.09		
3.83	1.06		
0.91	1.04		
4.13	1.12		

The variables of importance in projection (VIP) were selected by PLS-DA with a threshold of >1.0. Bins found to have a fold change (FC) > |1.5| were considered significant.

#### 3.2.4 Quantified metabolites

The metabolite identities of significant bins were confirmed using the two-dimensional (^1^H-^1^H JRES and COSY) NMR spectral data, and comparison to the pure compound spectral libraries, followed by absolute quantification (μmol/L). Metabolites that are significantly increased, based on PLS-DA VIP > 1.0, in the HIV+ group compared to the controls are lactate (bins 4.09, 1.35, 4.11, 1.33, 4.13), myo-inositol (bins 3.61, 3.31, 3.53, 3.29), 1,2-propanediol (bins 4.07, 1.13), and glutamine (bin 2.49). The significantly decreased metabolites, based upon PLS-DA VIP > 1.0, in the HIV+ group comparatively, are glucose (bins 3.25, 3.49, 3.41, 3.51, 5.25, 3.39, 3.23, 3.43, 3.83), 3-hydroxybutyrate (bin 2.39), acetoacetate (bin 2.29), and 2-hydroxybutyrate (bin 0.91). Identification and quantification of the significant bins, also selected by fold change, revealed that the metabolites that are increased in the HIV+ group compared to the controls are 1,2-propanediol (bins 4.07, 1.13), myo-inositol (bins 3.61, 3.31, 3.29), and lactate (bin 4.09), while acetoacetate (bins 2.29) is decreased.

**Figure 2:**
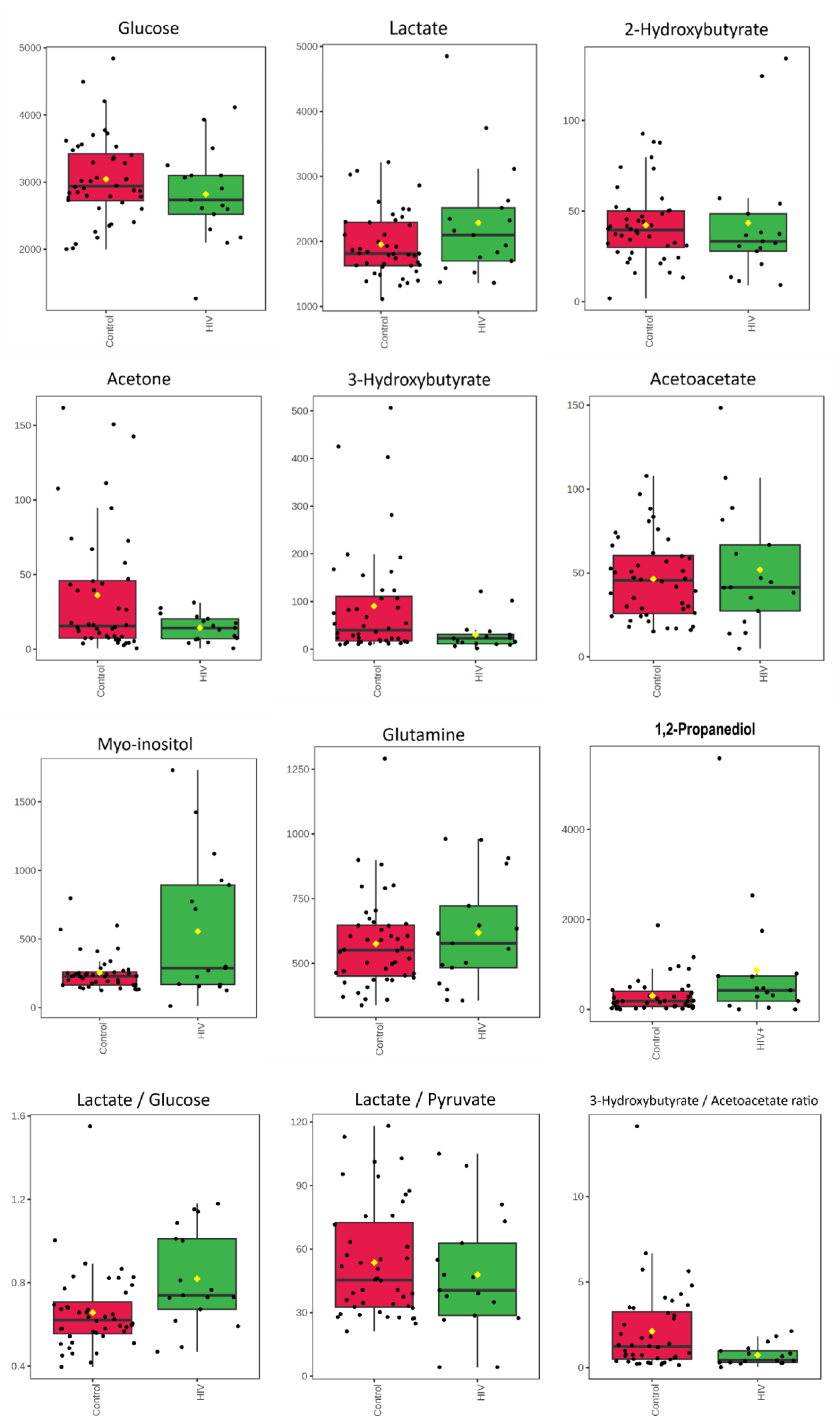
Boxplots for concentrations (μmol/L) of CSF metabolites characterizing HIV—glucose, lactate*, 2-hydroxybutyrate, acetone*, 3-hydroxybutyrate*, acetoacetate, myo-inositol*, glutamine, and 1,2-propanediol* as well as metabolite ratios: lactate/glucose*, lactate/pyruvate, and 3-hyrdorxybutyrate/acetoacetate*. * Indicates the metabolites and ratios that had significant effect sizes (*d* > 0.5) when comparing the 17 HIV+ (green) and 43 control (red) samples.

The quantified metabolic data and metabolite ratios are presented visually as box plots (Figure 2) and quantitatively in Table 3. It is important to note that acetoacetate and 3-hydroxybutyrate occupied the same significant ^1^H-NMR spectral bin; however, upon concentration quantification, it was found that acetoacetate did not significantly differ between the two groups. As an additional evaluation of the potential importance of ketone bodies, the metabolite concentration of acetone was also calculated. Calculation of the Mann–Whitney *U*-test with *p* < 0.05 indicates that metabolites that are significantly increased in the HIV+ group comparatively include myo-inositol and 1,2-propanediol, while 3-hydroxybutyrate is decreased in the HIV+ group comparatively. Cohen’s *d* value is a measure of effect size, indicating the strength of the relationship between two variables in a population. Cohen’s *d*-value calculation indicates that the metabolites: Lactate, 1,2-propanediol, 3-hydroxybutyrate, acetone, and myo-inositol have significant effect sizes (*d* > |0.5|).

**Table 3:** Summary of statistical findings for quantified metabolites.

Metabolite	HIV			Controls			HIV vs. controls	
	Average conc. (μmol/L)	±	Standard deviation	Average conc. (μmol/L)	±	Standard deviation	*p*-value	*d*-value
Glucose	2818.01	±	690.94	3024.34	±	552.30	0.288	−0.352
Pyruvate	51.62	±	36.24	45.58	±	25.21	0.508	0.322
Lactate	2285.20	±	912.51	1923.85	±	469.05	0.246	0.521*
Lactate:pyruvate ratio	53.71	±	25.44	54.26	±	26.81	0.889	0.002
Lactate:glucose ratio	0.81	±	0.23	0.64	±	0.13	0.008*	0.791*
Glutamine	618.46	±	209.38	561.69	±	141.43	0.541	0.231
1,2-Propanediol	985.70	±	1433.04	303.95	±	377.23	0.007*	0.850*
Acetone	15.06	±	8.40	37.46	±	43.30	0.322	−0.590*
3-Hydroxybutyrate	30.49	±	32.44	93.61	±	117.75	0.017*	−0.594*
Acetoacetate	51.92	±	37.32	46.23	±	24.18	0.961	0.193
3-Hydroxybutyrate: acetoacetate ratio	0.73	±	0.60	2.20	±	2.58	0.030*	−0.633*
2-Hydroxybutyrate	43.48	±	35.06	43.35	±	19.92	0.305	0.013
Myo-inositol	590.64	±	509.20	244.82	±	107.37	0.049*	1.182*

The Mann–Whitney *U*-test was used to obtain *p*-values with *p* < 0.05 considered significant. Effect sizes are considered significant at *d* > 0.5. Conc., concentration.

In addition, the lactate:pyruvate ratio and 3-hydroxybutyrate:acetoacetate ratio were determined to assess redox status, at the cytoplasm and mitochondrial levels, respectively. The lactate:glucose ratio was also calculated to indicate flux through glycolysis, into lactate.

#### 3.2.5 Correlations among metabolites

Spearman’s rho correlations indicate significant associations among several of the VIP metabolites. The correlation results are given in the supplementary information section. Glucose significantly positively correlates with the metabolites lactate (*r* = 0.524; *p* = 0.035), 3-hydroxybutyrate (*r* = 0.539; *p* = 0.035), acetoacetate (*r* = 0.507; *p* = 0.040), and 2-hydroxybutyrate (*r* = 0.667; *p* = 0.012). Lactate also significantly positively correlates with the metabolites: acetone (*r* = 0.576; *p* = 0.031), acetoacetate (*r* = 0.701; *p* = 0.011), and 2-hydroxybutyrate (*r* = 0.652; *p* = 0.013). The 1,2-propanediol significantly positively correlates with acetone (*r* = 0.569; *p* = 0.039). 3-Hydroxybutyrate significantly positively correlates with acetone (*r* = 0.588; *p* = 0.031), glutamine (*r* = 0.713; *p* = 0.011), and 2-hydroxybutyrate (*r* = 0.586; *p* = 0.027). The 2-hydroxybutyrate significantly positively correlates with acetone (*r* = 0.691, *p* = 0.012). Glutamine significantly positively correlates with acetone (*r* = 0.800; *p* = 0.010) and 2-hydroxybutyrate (*r* = 0.696; *p* = 0.011). Myo-inositol significantly positively correlates with glutamine (*r* = 0.653; *p* = 0.016).

#### 3.2.6 Correlations of metabolites with clinical features

Several significant associations were found between the VIP metabolites and clinical variables. Glucose significantly positively correlated with fever (*r* = 0.656; *p* = 0.014). Lactic acid significantly positively correlated with right hemiplegia (*r* = 0.704; *p* = 0.021) and CSF lymphocyte count (*r* = 0.661, *p* = 0.036). Myo-inositol significantly positively correlated with CSF protein (*r* = 0.629; *p* = 0.040), meningeal irritation (*r* = 0.678; *p* = 0.034), and cough (*r* = 0.583; *p* = 0.034). 1,2-Propanediol significantly negatively correlates with age (*r* = −0.526; *p* = 0.043). 3-Hydroxybutyric acid significantly correlated with viral count (*r* = 0.784; *p* = 0.032), left hemiplegia (*r* = −0.680; *p* = 0.029), decreased consciousness (*r* = −0.627; *p* = 0.021), and fever (*r* = −0.627; *p* = 0.047). Acetoacetate was significantly negatively correlated with a urinary tract infection (*r* = −0.631; *p* = 0.017). Glutamine significantly correlated with viral count (*r* = 0.825; *p* = 0.026), meningeal irritation (*r* = 0.648; *p* = 0.044), infarctions (*r* = −0.584; *p* = 0.034), and vomiting (*r* = 0.519; *p* = 0.045). 2-Hydroxybutyric acid significantly correlated with CSF protein (*r* = 0.738; *p* = 0.021), meningeal irritation (*r* = 0.608; *p* = 0.048), right hemiplegia (*r* = 0.657; *p* = 0.034), left hemiplegia (*r* = −0.606; *p* = 0.045), deceased (*r* = 0.492; *p* = 0.047), and fever (*r* = 0.601; *p* = 0.021).

#### 3.2.7 Correlations among clinical features

Several significant associations were found among the clinical variables. CD4 count positively correlated with ART exposure (*r* = 0.762; *p* = 0.030), infarctions (*r* = 0.633; *p* = 0.049), left hemiplegia (*r* = 0.866; *p* = 0.015), and gastroenteritis (*r* = 0.612; *p* = 0.045). Age significantly correlated with seizures (*r* = 0.631; *p* = 0.021) and TB contact (*r* = 0.669; *p* = 0.014). Raised intracranial pressure significantly correlated with hydrocephalus (*r* = 1.000; *p* = 0.001). Hydrocephalus significantly negatively correlated with fever (*r* = −0.535; *p* = 0.045). Meningeal irritation significantly correlated with vomiting (*r* = 0.671; *p* = 0.035) and TB contact (*r* = 0.810; *p* = 0.009). Infarctions significantly correlated with left hemiplegia (*r* = 0.674; *p* = 0.030) and decreased consciousness (*r* = 0.679; *p* = 0.019). Right hemiplegia significantly correlated with deceased status (*r* = 1.000; *p* = 0.001) and gastroenteritis (*r* = 1.000; *p* = 0.001). Gastroenteritis also significantly correlated with deceased status (*r* = 0.685; *p* = 0.009). Left hemiplegia significantly correlated with decreased consciousness (*r* = 1.000; *p* = 0.001) and fever (*r* = −0.674; *p* = 0.030). Diarrhea significantly correlated with gastroenteritis (*r* = 0.561; *p* = 0.009). Seizures significantly correlated with TB contact (*r* = 0.707; *p* = 0.011), cough (*r* = −0.709; *p* = 0.009), and pneumonia (*r* = −0.522; *p* = 0.045). Cough significantly correlated with TB co-infection (*r* = 0.522; *p* = 0.045) and pneumonia (*r* = 0.522; *p* = 0.045). Erythrocyte count significantly correlated with a urinary tract infection (*r* = 0.899; *p* = 0.001). Gastroenteritis significantly correlated with leukocyte count (*r* = 0.911; *p* = 0.001), lymphocyte count (*r* = 0.925; *p* = 0.001), CSF protein levels (*r* = 0.631; *p* = 0.045), and CSF glucose levels (*r* = −0.506; *p* = 0.047).

## 4 Discussion

Despite the relatively low prevalence of HIV co-infection among pediatric meningitis suspects in the hospital setting (Solomons et al., 2016), this study provides a rare opportunity to investigate the influence of HIV on the CNS compartment in perinatally infected children. The brain microenvironment is metabolically unique due to the strict regulation of metabolite exchange by the BBB. Regulation of brain metabolism is crucial, especially during early life, when energy demands for brain growth and plasticity are exceedingly high and can account for up to 44% of the metabolic rate and substrate requirements of the body (Oyarzábal et al., 2021). Fine-tuning metabolism in the brain is essential for maintaining energy homeostasis, supporting neuronal functions, protecting against oxidative stress, and facilitating communication within the nervous system. The findings of our study show the complex metabolic dynamics within the CNS of HIV-infected children, and the altered neurometabolism due to the aberrations of the underlying cellular processes induced by HIV infection. By delineating potential pathways of metabolic flux and cellular interactions, we aim to elucidate the changes in the neuroenergetics of pediatric HIV patients, which subsequently lead to the associated neurocognitive symptoms.

### 4.1 Perturbed neuroenergetics in HIV

**Figure 3:**
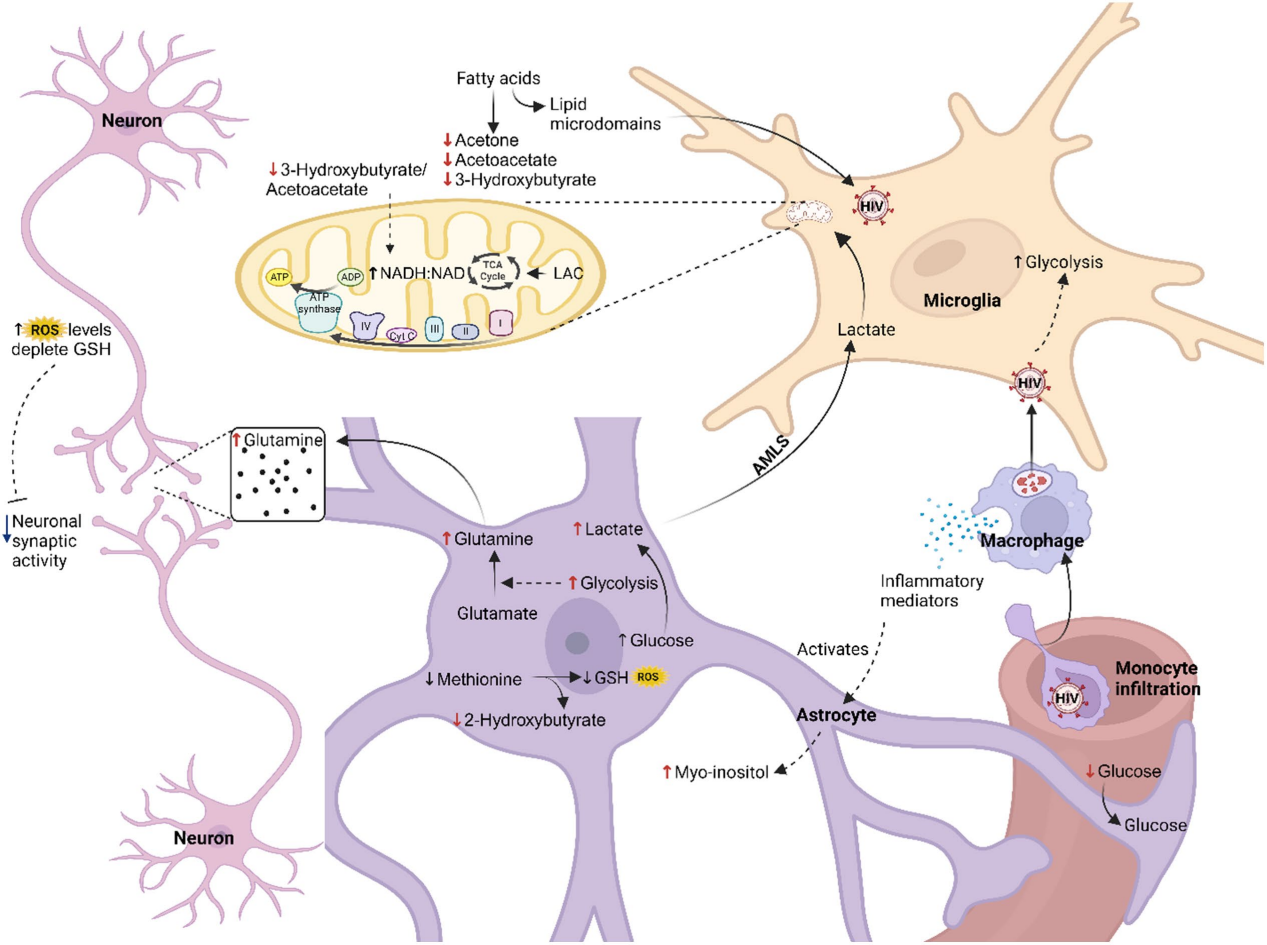
Hypothesized metabolic model of perturbed neuroenergetics in CSF samples collected from pediatric patients with HIV. AMLS, astrocyte-microglia lactate shuttle; LAC, lactate; ROS, reactive oxygen species; GSH, reduced glutathione (created with BioRender.com).

In this study, we found decreased levels of glucose and increased levels of lactate in the CSF of the HIV-infected participants, comparatively. Individually, these two metabolites were not statistically significant; however, the lactate:glucose ratio (Table 3) in the HIV+ group (0.81 ± 0.23) was significantly higher (*p* = 0.008) than the controls (0.64 ± 0.13), indicating an elevated flux through glycolysis toward lactate production (i.e., higher rate of glycolysis). Current literature suggests increased glycolysis in all HIV-infected cells, including microglia and infiltrating immune cells, which promotes the production and secretion of immune mediators that activate astrocytes and increase glycolysis in this cell type. The lactate:pyruvate ratios, however, were unchanged in the HIV+ group (53.71 ± 25.44) compared to the controls (54.26 ± 26.81), suggesting unaltered cytosolic NADH:NAD ratios. However, an increased mitochondrial redox potential [significantly lower (*p* = 0.030) 3-hydroxybutyrate:acetoacetate ratios in the HIV+ group (0.73 ± 0.60) compared to the controls (2.20 ± 2.58)] indicates elevated mitochondrial activity in the HIV+ group. As there is a persistent activation of the immune response in HIV, increased mitochondrial activity is likely localized to the microglia, as astrocytes are predominantly glycolytic (Magistretti and Allaman, 2018). However, a calcium overload in the neurons, induced by astrocyte dysfunction and oxidative damage, also likely triggers increased tricarboxylic acid (TCA) cycle activity. Significantly decreased ketones (acetone and 3-hydroxybutyrate) in the HIV+ group in this study are consistent with the findings of Keram et al. (2021) and Deme et al. (2022), but in contrast to the results of Cassol et al. (2014). The significantly elevated (*p* = 0.049) myo-inositol levels (590.64 ± 509.20) detected in the CSF of the HIV+ group, compared to the controls (244.82 ± 107.37) is indicative of astrocyte activation. While the exact mechanism for cellular interactions cannot be ascertained from this study alone, we hypothesize that astrocyte-derived lactate is being shuttled into the microglial cells [astrocyte–microglial lactate shuttle (AMLS); Mason, 2017] where it is utilized for energy production to support the persistent immune response known to occur in HIV patients. Hence, the increased flux of neuroenergetics may be channeled via astrocytes to microglia, inhibiting the usual energy shuttle to the neurons. This reduced flux of neuroenergetics toward the neurons could be due to a host-induced neuroprotective mechanism preventing oxidative damage to neurons. It is well known that HIV viral replication yields high amounts of reactive oxygen species (ROS), which subsequently leads to a depletion of intracellular reduced glutathione (GSH) (Perl and Banki, 2004). Re-synthesis of GSH consumes methionine, eventually depleting this metabolite, which, in turn, impairs GSH production, as is demonstrated by the reduced concentrations of 2-hydroxybutyrate in the HIV+ group comparatively, which forms as a by-product of this biosynthetic pathway. Reduced neuronal activity, evidenced by the higher levels of glutamine in the CSF of the HIV+ group comparatively, agrees with the reduced neurocognitive activity associated with HAND (Gleichmann and Mattson, 2011; Irollo et al., 2021). Figure 3 illustrates our hypothesized metabolic model, based on these results, of the perturbed neuroenergetics associated with the persistent immune activation in the brain of pediatric HIV patients.

### 4.2 Support of our hypothesized model from the literature

In this pediatric HIV-infected cohort, we observed elevated glycolytic pathway activity indicated by the high lactate:glucose ratio. Abnormalities in glucose metabolism are a well-documented occurrence in HIV participants (Sinharay and Hammoud, 2019; Deme et al., 2022). Increased glycolytic rate in virally suppressed adults and perinatally infected children correlates with worsening cognitive status (Dickens et al., 2015; Kaur et al., 2020). It is likely that the increased glycolytic rate, associated with the decline in cognitive ability in virally suppressed patients, is due to continued immune activation in the CNS compartment (Deme et al., 2022). Immune activation coupled with high biosynthetic and energetic demands requires extensive metabolic reprogramming (Shehata et al., 2017; Caslin et al., 2021). In conjunction with increased nutrient uptake, there is a considerable rise in glycolysis, despite sufficient oxygen levels, which is then used to fuel the biosynthetic pathways that produce the nucleotides and precursors for lipid and amino acid biosynthesis, which, in turn, allow for robust cell growth and proliferation (Shehata et al., 2017).

Clerc et al. (2019) indicated that glucose metabolism impacts the vulnerability of CD4+ T cells to HIV infection. The glucose transporter (GLUT-1) is upregulated on HIV-infected CD4+ T cells and required for cellular activation, the metabolic shift from oxidative phosphorylation (OXPHOS) to aerobic glycolysis, and consequently its effector functions (Palmer et al., 2016; Shehata et al., 2017; Clerc et al., 2019). Furthermore, high glucose uptake increases the expression of CXCR4 and CCR5, co-receptors required for HIV entry into a cell (Palmer et al., 2016; Clerc et al., 2019). This vulnerability may also partly be attributed to the activation of the mechanistic target of the rapamycin (mTOR) pathway as mTOR-complex 1 (mTORC1) upregulates CCR5 expression, while mTOR-complex 2 (mTORC2) leads to the induction of HIV transcription (Palmer et al., 2016; Shehata et al., 2017; Clerc et al., 2019). The HIV protein transactivator of transcription (tat) hyperactivates mTORC1 activity (Shehata et al., 2017). Moreover, HIV exacerbates the glycolytic shift in activated T cells and macrophages by upregulating the expression of glycolytic enzymes including glucose-6-phosphate dehydrogenase (G6PDH), hexokinase (HK), and pyruvate kinase M2 (PKM2) to highjack the cellular processes for virion production (Barrero et al., 2013; Shehata et al., 2017; Deme et al., 2022). It is possible that dysregulation of immune cell metabolism, including excessive uptake of glucose, might induce hyperactivation of the immune system and contribute to HIV pathogenesis (Palmer et al., 2016; Deme et al., 2022).

This study found further evidence of altered neuroenergetics indicated by the decreased levels of ketone bodies in the CSF of this HIV+ group. During prolonged fasting periods, diabetes, and neonatal or pre-weaning development, the brain becomes dependent on ketone bodies for energy production (Deme et al., 2020). The lower 3-hydroxybutyrate:acetoacetate ratio indicates elevated mitochondrial activity in the HIV+ group. As there is a persistent activation of the immune response in HIV, increased mitochondrial activity is likely localized to the microglia as astrocytes are predominantly glycolytic (Magistretti and Allaman, 2018). Furthermore, in HIV-infected cells acetyl-CoA is also directed toward fatty acid synthesis, via increased fatty acid synthase activity, thereby further reducing the downstream formation of ketone bodies (Kulkarni et al., 2017). Fatty acid synthase produces longer chain fatty acids, which are required for the generation of lipid micro-domains essential for viral budding or to produce fatty acyl adducts, which are necessary for post-translational modification of viral proteins such as Env, Gag, and Nef (Kulkarni et al., 2017; Deme et al., 2022). Moreover, Vpr reduces transcription of acetyl-CoA acetyltransferase in macrophages, which encodes for mitochondrial acetoacetyl-CoA thiolase, the enzyme that converts two units of acetyl-CoA to acetoacetyl-CoA, for ketone body production (Barrero et al., 2013). Therefore, successful suppression of viral replication by ART use has been shown to normalize ketone body serum levels (Deme et al., 2022). Beyond being substrates for neuronal ATP synthesis, ketone bodies have a variety of paracrine and autocrine signaling functions that alter gene expression, lipid metabolism, neuronal function, and metabolic rate (Newman and Verdin, 2017). Tissue culture experiments found that the treatment of neurons with ketone bodies protects these cells against tat-induced oxidative stress and calcium-induced hyperexcitability (Kim et al., 2010; Hui et al., 2012; Garcia-Rodriguez and Gimenez-Cassina, 2021). Ketone body utilization also produces high levels of ATP and can stimulate mitochondrial biogenesis (Hui et al., 2012). Considering the various regulatory and protective functions that ketone bodies are known to exert in the CNS, dysregulation of ketone body metabolism in children infected with HIV is likely to contribute to the pathology of HIV infection and the brain abnormalities observed in this population. The long-term effects of aberration of ketone body metabolism, as it occurs during critical developmental stages, need further investigation. Our study found reduced levels of 2-hydroxybutyrate in the HIV+ group comparatively, indicating exhaustion of the antioxidant systems. HIV replication exacerbates oxidative damage in the CNS compartment by reducing intracellular GSH levels and modulating the transcription of several enzymes involved in GSH metabolism (Porcheray et al., 2006). When GSH becomes depleted, methionine and cysteine are both directed toward its re-synthesis through the sequence of reactions, methionine > cystathionine > cysteine > GSH (Lertratanangkoon et al., 1996). During the conversion of cystathionine to cysteine, 2-ketobutyrate is produced and then reduced to 2-hydroxybutyrate (Legault et al., 2015). Other studies have reported methionine deficiencies in ART-naïve adults and children with HIV, which would also result in the reduced formation of 2-hydroxybutyrate, but these levels are corrected with ART (Kaur et al., 2020; Deme et al., 2022). Our findings indicate an inability to control oxidative stress levels in the CNS compartment of children with HIV.

ROS can also function as signaling molecules and contribute to neuronal development and function. ROS mediates various functions within the nervous system via the glial cells and glial-neuron interactions (Oswald et al., 2018). Myo-inositol, elevated in the CSF of the HIV+ group in our study, is a marker of astrocyte activation (Cassol et al., 2014; Dickens et al., 2015; Kaur et al., 2020). Gliosis, a characteristic of the chronic neuroinflammation induced by HIV, persists despite ART (Kaur et al., 2020). Previous studies have associated higher CSF myo-inositol levels with a decline in cognitive function, even in virally suppressed HIV patients (Ernst et al., 2003; Harezlak et al., 2011; Cassol et al., 2014; Williams et al., 2021a). Other studies have also confirmed abnormalities in myo-inositol metabolism in children infected with HIV, particularly in the left frontal brain region (Kaur et al., 2020). The intracellular concentrations of myo-inositol are influenced by several processes, including uptake, biosynthesis, recycling from derivatives, degradation, and efflux (Croze and Soulage, 2013). Considering the latter, increased efflux due to cell swelling results in the depletion of intracellular myo-inositol. This decrease in intracellular myo-inositol levels impairs phosphoinositide (PI) signaling, disrupting the intracellular calcium balance, resulting in neuronal hyperexcitability, and causing deficiencies in neurotransmitter release and ion conductivity (Croze and Soulage, 2013).

We hypothesize that alterations in PI turnover contribute to the impairment of neuronal activity. Patients with moderate-to-severe HAND present with reduced synaptic transmission (Irollo et al., 2021). Disruption of PI signaling is associated with reduced Na^+^/K^+^-ATPase activity, leading to impaired nerve conduction (Lang et al., 1998; Fisher et al., 2002; Croze and Soulage, 2013). The disruption of calcium signaling and homeostasis, induced by altered PI signaling, is augmented by high ROS levels that cause oxidative damage to the mitochondria reducing its buffering capacity, thereby increasing the susceptibility to excitotoxicity (Gleichmann and Mattson, 2011). In response, neurons can initiate a homeostatic adaptation called synaptic scaling (Green et al., 2019), which aims to rebalance excitatory and inhibitory synaptic transmission, typically resulting in a de-potentiation of excitatory synapses and a heightened inhibitory transmission in HAND models (Gleichmann and Mattson, 2011; Green et al., 2019). Support for this hypothesis comes from the viral protein, tat, which has been shown to alter intracellular calcium levels via the inositol-1,4,5-trisphosphate (IP3) receptor (IP3R) in rat hippocampal neurons (Cotto et al., 2019). With chronically high ROS levels, energy production will eventually diminish, and neurons will become prone to excitotoxicity (Gleichmann and Mattson, 2011).

Accumulation of glutamine in the CSF of our HIV+ cohort suggests altered neural activity. Astrocytes regulate glutamatergic and GABAergic neurotransmission through the activity of the glutamate-glutamine cycle, which can be dysregulated by both a pro-inflammatory environment and HIV proteins (Chaudhry et al., 2002; Vazquez-Santiago et al., 2014; Bairwa et al., 2016; Mason, 2017; Irollo et al., 2021). The balanced cycling of glutamate and glutamine is essential for normal neuronal function (Bairwa et al., 2016). Several studies have reported significantly decreased GABAergic markers in brain samples from HIV-infected participants with and without HAND and have found correlations with neuroimmune markers in the brain (Borjabad and Volsky, 2012; Irollo et al., 2021). GABAergic signaling is involved in neuronal proliferation, migration, differentiation, preliminary circuit-building, the formation of interstitial neurons in the white matter, and the development of oligodendrocytes (Wu and Sun, 2015). Disruption of GABAergic signaling may be a contributing factor to the white matter abnormalities often associated with HIV infection (Irollo et al., 2021). Moreover, abnormal excitatory glutamate neurotransmission has long been reported in HAND patients (Irollo et al., 2021). Glutamatergic neurotransmissions are involved in synaptic plasticity, learning and memory, and mood regulation (Madeira et al., 2018). Therefore, the long-term consequences of altered glutamate-glutamine cycling could be severe and possibly contribute to the development of HAND (Madeira et al., 2018). We hypothesize that aberration of myo-inositol metabolism and high ROS levels could contribute to the reduced neuronal activity observed in HAND (Borjabad and Volsky, 2012; Irollo et al., 2021), by dysregulating calcium homeostasis, leading to activation of compensatory mechanisms that function to counteract excitotoxicity (Green et al., 2019). The result is reduced rates of glutamine uptake in the neurons and subsequently elevated glutamine in the CSF of the HIV+ group.

In our study, the positive correlation of glutamine with 2-hydroxybutyrate suggests that glutamine may be increased due to a compensatory mechanism that lowers neural synaptic activity to reduce ROS production, reducing the requirement for GSH synthesis. 2-Hydroxybutyric acid also correlated with fever, CSF protein, and meningeal irritation, suggesting that high ROS levels cause damage to the BBB. The increased glycolytic rate in astrocytes would also drive the glutamate to glutamine conversion in these cells (Mason, 2017), possibly explaining the correlation of glutamine with viral count. Glutamine also correlated with meningeal irritation and vomiting, which may be another consequence of increased viral replication and astrocyte activation as HIV is known to disrupt the integrity of the BBB (McArthur and Johnson, 2020). Astrocytes are critical structural components of the BBB; hence, astrocyte activation and disruption of their normal cellular function diminish the integrity of the BBB, leading to meningeal irritation, indicated by the correlation of this clinical feature as well as CSF protein levels with myo-inositol. The positive correlation of myo-inositol with glutamine may confirm that altered PI signaling interferes with neuronal activity. Further investigation is required to confirm or refute this hypothesis.

As astrocytes form an integral part of the BBB, they act as a cellular barrier between the blood and neurons (Pellerin, 2010). Astrocytes play a crucial role in supporting neuronal activity, not just by recycling neurotransmitters but also because the uptake of glutamate stimulates an increase in glucose uptake, glycolysis, and lactate production, which are released into the extracellular space, in response to synaptic activity, in order accommodate the energy demands of these neurons. This model has been termed the astrocyte–neuron lactate shuttle (ANLS) hypothesis (Mason, 2017). Under conditions of sustained physiological challenges, such as in HIV, the astrocyte–microglia lactate shuttle (AMLS) hypothesis suggests that brain energy metabolism shifts away from neurons to provide lactate to activated microglia in support of the immune response (Mason, 2017). Lactate modulates several key microglia features, including proliferation, migration, and phagocytosis (Monsorno et al., 2022). Within microglia, lactate enters the TCA cycle and electron transport chain to produce ATP and the ROS necessary for eliminating invading pathogens (Mason, 2017; Monsorno et al., 2022). Further investigation is required to confirm or refute this AMLS hypothesis.

In our study, an additional source of lactate would be via the increased concentrations of 1,2-propanediol, also previously associated with HAND in adults infected with HIV (Cassol et al., 2014). 1,2-propanediol, found to be elevated in this HIV+ group, functions as a solvent in a variety of intravenously administered medications and may originate from higher medication usage in the HIV+ group comparatively. Endogenous sources, however, include dihydroxyacetone phosphate metabolism or acetone breakdown (Ruddick, 1972). Viral protein R (Vpr) reduces the expression of triosephosphate isomerase (TPI) in macrophages to direct more glucose to the PPP (Barrero et al., 2013). The downregulation of TPI in the frontal cortex of HIV patients is associated with neurodegeneration (Zhou et al., 2010). This leads to the accumulation of dihydroxyacetone phosphate, which is eventually metabolized to 1,2-propanediol.

### 4.3 Correlations

The correlations between the metabolites improve our understanding of the interplay between the discussed pathways. Glucose correlated with lactate and metabolites related to ketone body metabolism. We expected this as all these metabolic pathways are directed toward energy production, intersecting at the TCA cycle. Furthermore, mTOR activity regulates both glycolysis and ketogenesis. The positive correlation between glucose and metabolites of ketone body metabolism, which were both lower in the HIV+ group, suggests the utilization of these metabolites to meet the increased energy demand of an HIV-infected brain. In the study by Deme et al. (2022), higher serum methionine levels, and by extension higher 2-hydroxybutyrate levels, correlated with higher CD4+ T counts in treatment-naïve adults with HIV, indicating its role in the control of viral replication. Hence, this might also explain the positive correlation of glucose with 2-hydroxybutyrate as higher rates of viral replication would lead to higher ROS production, decreasing methionine levels, increasing activation of the glycolytic pathway, and decreasing CSF glucose levels (Lertratanangkoon et al., 1996; Shehata et al., 2017).

Reduced CSF glucose levels are an indicator of increased immune activation in the CNS compartment, supporting the positive correlation between glucose and fever (Shehata et al., 2017). The positive correlation of lactate with the CSF lymphocyte counts suggests that this metabolite originates from immune activation or viral replication (Pajkrt et al., 2016). 3-Hydroxybuturate positively correlated with acetone, fever, and viral count, suggesting that ketone body metabolism supports viral replication by increasing the activity of the TCA cycle, which is known to promote virion production (Barrero et al., 2013). The positive correlations of 3-hydroxybutyrate and acetone with glutamine and 2-hydroxybutyrate suggest increased viral replication results in oxidative stress, which subsequently leads to increased GSH synthesis, marked by elevated 2-hydroxybutyrate and the initiation of a protective mechanism that reduces neural activity, increasing extracellular glutamine (Lertratanangkoon et al., 1996; Gleichmann and Mattson, 2011; Irollo et al., 2021). Similarly, the positive correlation of acetone with 2-hydroxybutyrate may signify an increased requirement for GSH due to oxidative stress induced by HIV viral replication (Perl and Banki, 2004).

Alterations in brain energy metabolism are apparent already in the early stages of disease following HIV infection and do not fully normalize even after successful viral suppression (Deme et al., 2020). Our research group and others have observed that HIV induces changes to the host’s cell metabolism to hyperactivate glycolysis, augments lactate production, and increases the flux through the PPP, decreasing β-oxidation of long-chain fatty acids and increasing amino acid catabolism (Deme et al., 2022). Furthermore, similar metabolic changes in the brain’s energy metabolism have been associated with several other neurodegenerative diseases, including HAND, and also resemble age-related mechanisms of pathogenesis (Cassol et al., 2014; Deme et al., 2020).

### 4.4 Limitations and strengths

Several limitations exist in this study, such as the limited number of HIV+ samples available, a control group that was not entirely healthy, and the absence of a validation cohort. However, the importance is to recognize that these limitations are not easily surmountable given that the study’s focus is on a pediatric patient population and that CSF samples, rare to obtain, were used. Few studies have investigated CSF in pediatric HIV cases, making this study unique and its findings extremely valuable. As this study was holistic and exploratory in nature, we subsequently identified several new metabolite markers for HIV in a pediatric population, which not only provides numerous new biochemical hypotheses to direct future studies but also supports existing hypotheses or conclusions made by other groups. The hypothesized metabolic model proposed here requires further scientific engagements to support or refute our hypotheses.

## 5 Conclusion

The results of this study confirm previous findings that HIV dysregulates cellular metabolic processes, including upregulating glycolysis, dysregulating ketone body metabolism, and increasing ROS production. These changes relate to the increased metabolic demand associated with immune activation and high viral replication. We hypothesize that neuroinflammation alters astrocyte-neuron communication and lowers neuronal activity in children infected with HIV, which, in turn, may contribute to the neurodevelopmental delay often observed in this population.

## Data availability statement

The raw data supporting the conclusions of this article will be made available by the authors, without undue reservation.

## Ethics statement

The studies involving humans were approved by the Health Research Ethics Committee (HREC) of Stellenbosch University, Tygerberg Hospital (ethics approval no. N16/11/142), and the HREC of the North-West University (NWU), Potchefstroom campus (ethics approval no. NWU-00063-18-A1). The studies were conducted in accordance with the local legislation and institutional requirements. Written informed consent for participation in this study was provided by the participants’ legal guardians/next of kin.

## Author contributions

AT: Data curation, Formal analysis, Investigation, Methodology, Writing – original draft. DL: Formal analysis, Funding acquisition, Resources, Supervision, Writing – review & editing. MW: Formal analysis, Resources, Supervision, Writing – review & editing. RS: Data curation, Investigation, Writing – review & editing. SM: Conceptualization, Data curation, Formal analysis, Methodology, Project administration, Resources, Supervision, Writing – review & editing.
