# Supplementary material for: 1H-NMR metabolomics investigation of CSF from children with HIV reveals altered neuroenergetics due to persistent immune activation
Source: Front Neurosci. 2024 Apr 30;18:1270041. doi: 10.3389/fnins.2024.1270041 (PMC11091326; doi:10.3389/fnins.2024.1270041)
Supplement: Supplementary file 1 [file Image_1.pdf]

Supplementary Material

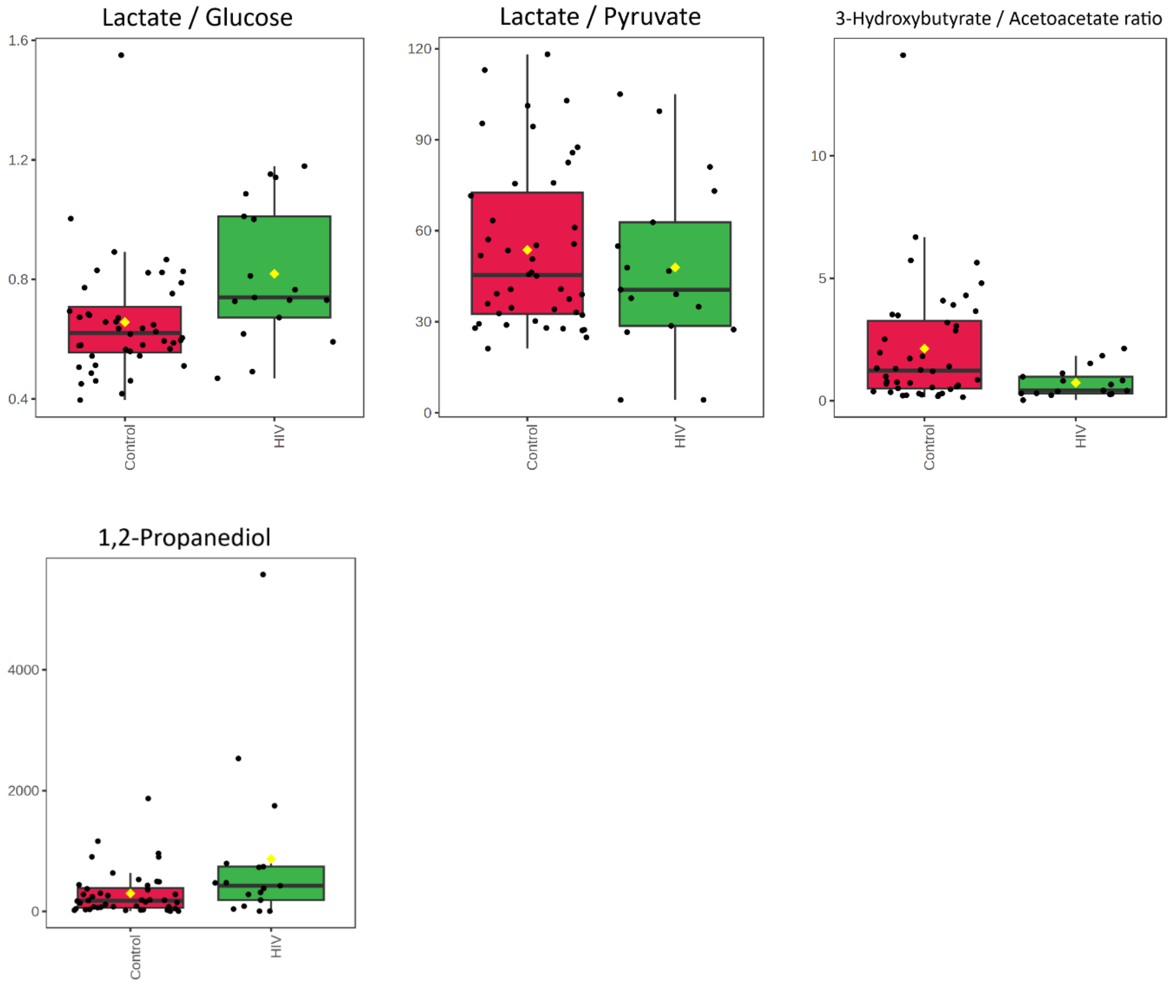

Supplementary Figure 1. Boxplots for all other quantified metabolites and ratio boxplots.
